# Identification of the Core MicroRNAs and Potential Molecular Mechanismsin Sarcoidosis Using Bioinformatics Analysis

**DOI:** 10.3389/fmolb.2021.644232

**Published:** 2021-05-13

**Authors:** Yuan Cao, Hua Zhang, Lulu Zheng, Qiao Li

**Affiliations:** ^1^Department of Pulmonary and Critical Care Medicine, The Second Affiliated Hospital of Xi’an Jiaotong University (Xibei Hospital), Xi’an, China; ^2^Department of Respiratory Medicine, Zhangjiakou First Hospital, Zhangjiakou, China; ^3^Clinical Laboratory, The Affiliated Children Hospital of Xi’an Jiaotong University, Xi’an, China

**Keywords:** sarcoidosis, bioinformatics analysis, microRNA-126, VEGFA, NR3C1

## Abstract

Sarcoidosis is a systemic heterogeneous inflammatory disease; however, the etiology and pathogenesis of sarcoidosis are still unknown. Herein, we investigated the core microRNAs and potential molecular mechanisms in sarcoidosis. The DE-miRNAs were diagnosed using the LIMMA software package. DIANA-mirPath was employed to perform pathway and GO enrichment analysis of the DE-miRNAs. PPI networks and miRNA-target gene regulatory networks were used to obtain insight into the actions of DE-miRNAs. Expression of the hub genes along with miRNAs was validated in clinical specimens. Overall, 266 DE-miRNAs were screened. Among these DE-miRNAs, hsa-miR-144, hsa-miR-126, as well as hsa-miR-106a were the upmost upregulated miRNAs; hsa-miR-151-3p, hsa-miR-320d, and hsa-miR-324-3p were the top downregulated miRNAs. *NR3C1*, *ZBTB7A*, *NUFIP2*, *BZW1*, *ERGIC2*, and *VEGFA* were mapped as the most targeted hub genes in the upregulation of miRNAs, and *MCL1* and *SAE1* were the most targeted hub genes in the downregulation of miRNA. *VEGFA* and *NR3C1* were selected and potentially modulated by hsa-miR-20b, hsa-miR-126, and hsa-miR-106a. In sarcoidosis pathological tissue, hsa-miR-126 was highly expressed, and VEGFA and NR3C1 were overexpressed. In conclusion, our results revealed the dysregulation of hsa-miR-126 and a potential regulatory mechanism for pathogenesis in sarcoidosis.

## Introduction

Sarcoidosis is a systemic heterogeneous inflammatory disease that usually leads to a multisystemic granulomatous disorder (Iannuzzi et al., 2007; Jain et al., 2020). It can affect any organ; however, at least 90% of cases occur in intrathoracic lymph nodes, as well as the lungs (Ungprasert et al., 2019). The annual incidence of sarcoidosis changes with race and ethnicity and is highest in African Americans (17 to 35/100,000) and lowest in Asians (1/100,000). However, the incidence may be underestimated, as almost 50% of the sarcoidosis patients are asymptomatic, especially those in the early stage of the disease. Epidemiologic studies have reported that the maximum incidence of sarcoidosis occurs in female non-smokers with an average age of 30 (Jain et al., 2020). The characteristic pathologic lesion of pulmonary sarcoidosis is a non-necrotizing granuloma (Iannuzzi et al., 2007). The etiology and pathogenesis of sarcoidosis are still unknown, and genetic susceptibility, autoimmunity, and environmental factors are all considered to be involved in the development of sarcoidosis.

MicroRNAs (miRNAs) are small, single-stranded, non-coding RNAs approximately 22 nucleotides long, some of which have been discovered to perform significant modulatory functions in animals through targeting the messages of protein-coding genes for translational inhibition. Research has documented that changes in the expression levels of miRNAs participate in multiple pathologies, as well as diseases. A previous study evaluated the expression trend of multiple miRNAs in sarcoidosis, and selected miRNAs were identified. However, regulatory miRNAs, their target genes, and the correlated protein expression have seldom been explored (Pattnaik et al., 2020). In this regard, we examined the microarray data of the gene expression profile of GSE26409 by a series of biological informatics approaches. The potential target genes of the upmost up-regulated, as well as down-regulated miRNAs were prognosticated by miRTarBase, and their potential functions were evaluated by DIANA-mirPath. Then, a protein–protein interaction (PPI) and miRNA-gene modulatory network were established by Cytoscape. Moreover, we validated the expression status of these hub genes and their modulated microRNAs in sarcoidosis samples by immunohistochemistry (IHC) along with fluorescence *in situ* hybridization (FISH). Based on a gene chip array, we used biological information technology to reveal the potential pathway in the etiology of sarcoidosis and yield additional information for its diagnosis, prognosis and treatment.

## Materials and Methods

### Microarray Data

The microarray GSE26409 cohort was selected for further study and abstracted from the National Center for Biotechnology Information GEO (Gene Expression Omnibus) website^1^, which is available online. This data set was based on the GPL9040 platform (febit Homo Sapiens miRBase 13.0) and included 45 samples from sarcoidosis patients and 55 from normal controls.

### Screening for DE-miRNAs

First, normalization of the downloaded data was performed using the Normalize Between Array function in the Bioconductor R package “LIMMA” (available online: http://www.bioconductor.org/). Then, the unpaired Student’s *t*-test was employed in comparing the sarcoidosis group with the control group. The cut-off criterion for screening differentially expressed miRNAs (DE-miRNAs) were *p* < 0.05 along with | fold change (FC)| > 1. Additionally, the GEO2R online analytic tool based on GEO data platform was employed further to validate the DE-miRNAs.

### Prediction of Target Genes of DE-miRNAs

MiRTarBase (available online: http://mirtarbase.mbc.nctu.edu.tw/php/index.php) is extensively employed to search for miRNA target genes. The microRNA-target interactions in this database were experimentally validated. Herein, miRTarBase was employed to identify the target genes of DE-miRNAs.

### GO Annotation and KEGG Pathway Enrichment Analyses of DE-miRNAs

DIANA-mirPath (available online: https://microrna.gr/miRPathv3/) constitutes a miRNA pathway analysis web-platform that provides accurate statistics and at the same time accommodates advanced pipelines (Vlachos et al., 2015). The functional, as well as pathway enrichment of the candidate miRNAs were explored and annotated by mirPath v2.0 based on the database Tarbase v7. The GO (Gene Ontology) was annotated through the DIANA-mirPath online tool on the selected DE-miRNAs. The KEGG (Kyoto Encyclopedia of Genes and Genomes) pathway analysis of DE-miRNAs was also performed by using DIANA-mirPath. *p* < 0.05 signified statistical significance.

### PPI Network Integration

To analyze the connection among proteins, the identify target genes of the upmost 5 most up and downregulated DE-miRNAs were uploaded to the STRING data resource^2^, and the results were visualized in Cytoscape 3.7.1 (Damian et al., 2015). Furthermore, we screened the significant nodes or hub genes based on degree, and then the miRNA-hub gene networks were mapped with Cytoscape 3.7.1.

### Immunohistochemistry (IHC)

Immunohistochemistry was used to further validate the paraffin sections of mediastinal lymph node tissues from pulmonary sarcoidosis patients and tuberculosis patients (control group), which came from the Second Affiliated Hospital of Xi’an Jiaotong University, with ethical approval. All specimens were obtained by endobronchial ultrasound-guided transbronchial needle aspiration (EBUS-TBNA), and all the patients in the study group and the control group have been screened, and those with cardiovascular and cerebrovascular diseases, endocrine, infectious diseases, tumor diseases, etc., have been excluded. The diagnosis was made based on guidelines (Costabel and Hunninghake, 1999). The tissue paraffin sections were first deparaffinized with dimethylbenzene and ethyl alcohol for 15 min, repaired by boiling for 8 min in the repairing solution with EDTA (pH 9.0), digested for 10 min by 3% H_2_O_2_ at 37°C, washed with PBS solution and blocked for 30 min with 3% BSA at room temperature (RT). Afterward, the sections were overnight-incubated with the primary antibody (NR3C1, VEGFA) at 4°C (Abcam, Cambridge, United States). The next day, the tissues were inoculated with the horseradish-peroxidase (HRP) and incubated at RT for 60 min and incubated with diaminobenzidine (DAB) color liquid after washing. Afterward, the tissues were counterstained using Harris hematoxylin, 1% hydrochloric acid alcohol, and lithium carbonate blue sequentially, and then visualized using an optical microscope (10×, 40×, Nikon, Japan) after routine cleaning and drying.

### Fluorescence *in situ* Hybridization (FISH)

*In situ* hybridization was carried out on the clinical samples using a FISH kit (Servicebio, Wuhan, China). First, tissue paraffin sections were deparaffinated with dimethylbenzene and 100% ethyl alcohol for 15 min, repaired by boiling for 8 min, digested by protein kinase K at 37°C for 10 min, washed 2× with a saline-sodium citrate (2× SSC) solution and dried before hybridization. Then, the prepared tissues were prehybridized at 37°C for 60 min, and hybridized with a hsa-miR-126, hsa-miR-20b, or hsa-miR-106a oligodeoxynucleotide probe (Shenggong, Shanghai, China) in the hybridization solution at 37°C overnight in the dark. Hsa-miR-126 probe: 5′-FAM-UCGUACCGUGAGUAAUAAUGCG-FAM-3′ (Entrez Gene 574032, Xq26.2); hsa-miR-20b probe: 5′-FAM- CTACCTG CACTATGAGCACTTTG-FAM-3′ (Entrez Gene 406913, 9q3 4.3); hsa-miR-106a: 5′-FAM-ATCTGCACTGTCAGCACTTTA-FAM-3′ (Entrez Gene 406899, Xq26.2). The next day, the tissues were washed by 2× SSC solution and 3% BSA employed to block the tissues for 30 min at RT, then counterstained with anti-digoxigenin antibody and DAPI (4′,6-diamidino-2-phenylindole, dihydrochloride), all from Servicebio, and imaged using a fluorescence microscope (400×, Nikon, Japan) and measured using Image-J software at two to three fields after routine cleaning and drying. Mean fluorescence intensity (MFI) was calculated. All measured intensities were normalized to the respective channel in control tissues.

## Results

### Identification of DE-miRNAs and Prediction of Target Genes

**FIGURE 1:**
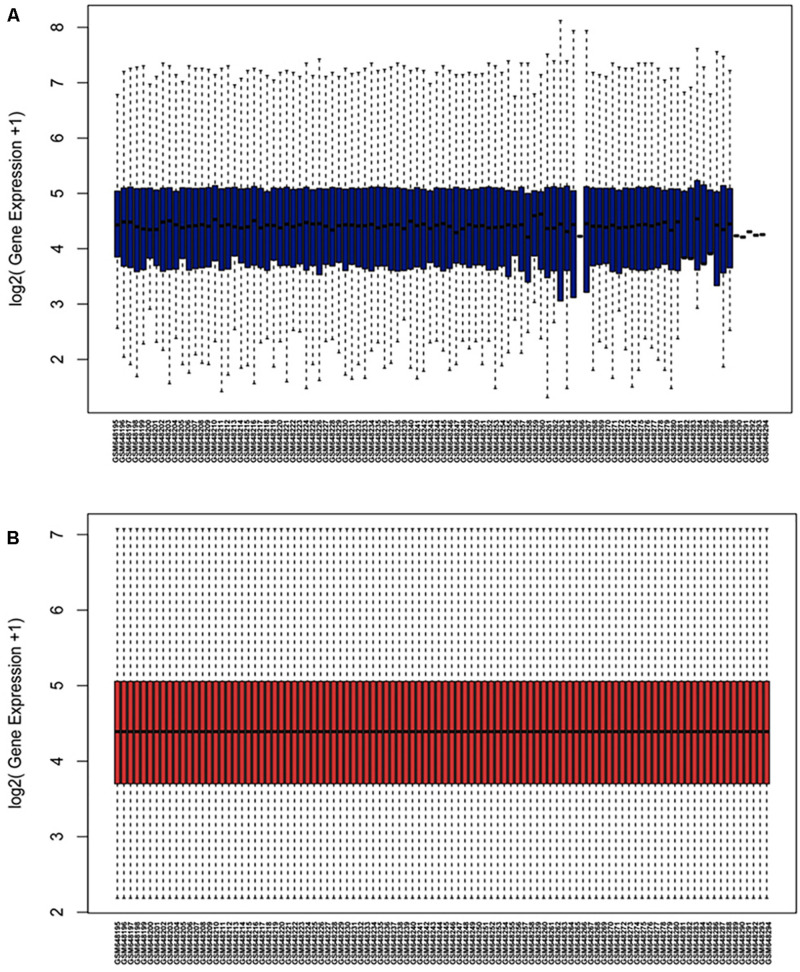
Normalization of dataset GSE26409 **(A)** raw data of GSE26409; **(B)** standardization of GSE26409.

**FIGURE 2:**
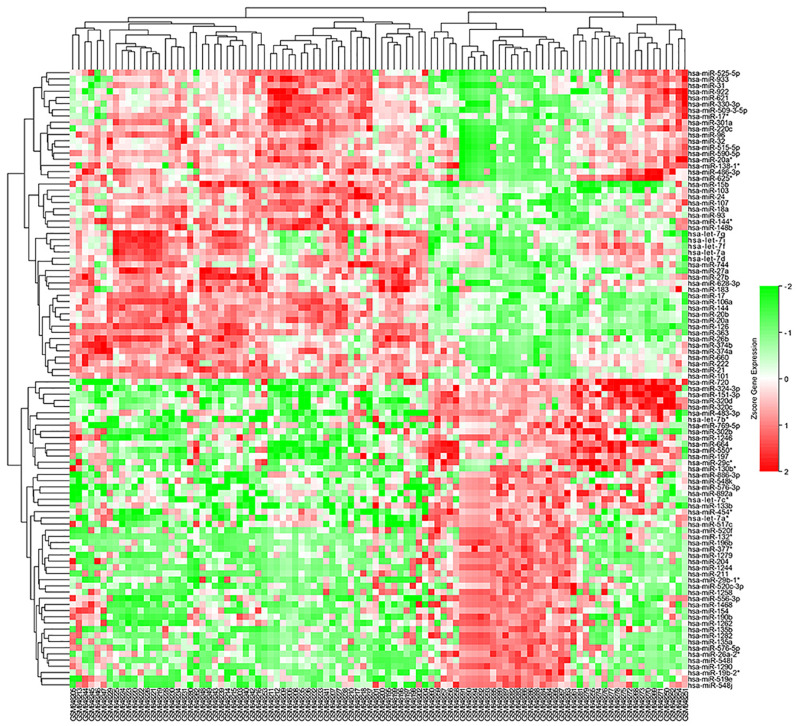
Heatmap of DE-miRNAs: DE miRNAs (differentially expressed miRNAs) in 45 cases of sarcoidosis and 55 cases of normal controls.

**FIGURE 3:**
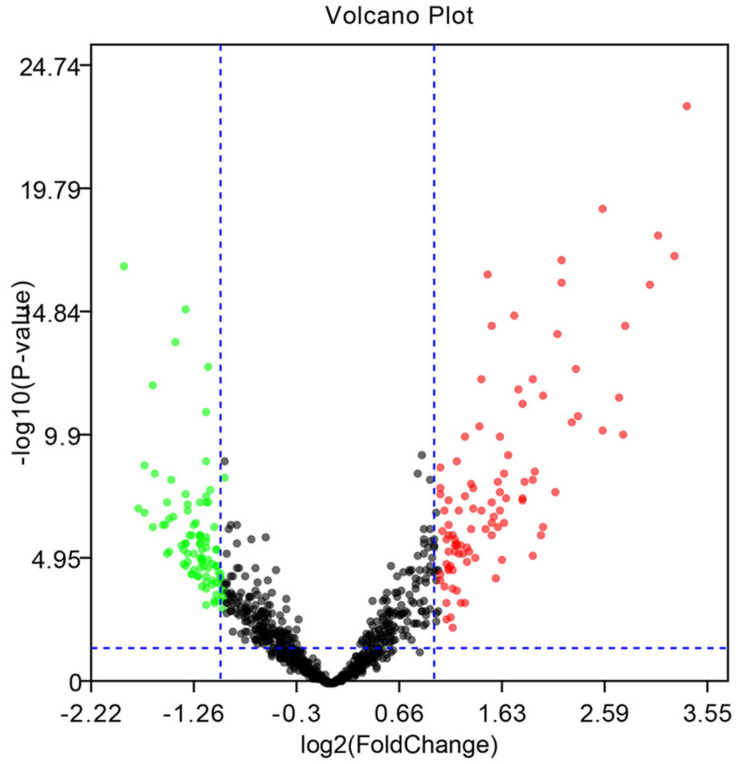
Volcano diagram of the DE-miRNAs. The black dots designate the genes with no remarkable difference. The red dots and green dots designate up-regulated and down-regulated genes selected according to | fold change| > 1.0 along with a corrected *p* < 0.05.

The microarray GSE26409 cohort was acquired from the GEO website, and this cohort consisted of 45 cases of sarcoidosis (GSM648250-GSM648294) and 55 normal controls (GSM648195-GSM648249). After normalization (Figure 1), processing of the data was conducted via unpaired *t*-test, with the cutoff criterion of *P* < 0.05 along with | log2FC| > 1). Overall, 266 DE-miRNAs were finally screened (Figures 2, 3), which constituted 132 and 134 upregulated and downregulated miRNAs, respectively. The top ten most upregulated, as well as downregulated miRNAs are indicated in Table 1. On the basis of the fold change (FC), hsa-miR-144, hsa-miR-126, and hsa-miR-106a were the upmost 3 upregulated miRNAs and hsa-miR-151-3p, hsa-miR-320d, and hsa-miR-324-3p were the upmost 3 downregulated miRNAs. For the three upregulated miRNAs, 580 potential target genes were investigated, and 543 genes were predicted for the 3 downregulated miRNAs via the miRTarBase platform.

**TABLE 1:** Top ten up-regulated and down-regulated differentially expressed miRNAs between sarcoidosis and normal control.

Row names(tT)	logFC	AveExpr	*t*	*P* Value	Adj. *P* Val	B	Regulated
hsa-miR-144	3.550863	9.313051	13.49568	9.40E-25	4.05E-22	45.78095	Up-Regulated
hsa-miR-126	3.320719	9.096936	13.12656	5.97E-24	1.72E-21	43.9572	Up-Regulated
hsa-miR-106a	2.530724	11.62744	11.24603	8.78E-20	1.90E-17	34.4828	Up-Regulated
hsa-miR-20b	3.063342	10.19364	10.80117	8.78E-19	1.52E-16	32.20948	Up-Regulated
hsa-miR-363	3.221189	10.27816	10.42804	6.08E-18	8.75E-16	30.29805	Up-Regulated
hsa-miR-15b	1.469123	13.24834	10.08452	3.62E-17	3.47E-15	28.53664	Up-Regulated
hsa-miR-103	2.156385	11.94484	9.951238	7.24E-17	6.25E-15	27.85326	Up-Regulated
hsa-miR-20a	2.986091	10.55733	9.923751	8.35E-17	6.55E-15	27.71235	Up-Regulated
hsa-miR-27a	1.718716	7.379181	9.366076	1.51E-15	1.00E-13	24.85707	Up-Regulated
hsa-miR-17	2.75136	11.48185	9.189665	3.75E-15	2.31E-13	23.95629	Up-Regulated
hsa-miR-151-3p	−2.12643	9.873969	−13.827	1.81E-25	1.56E-22	47.40578	Down-Regulated
hsa-miR-320d	−1.93278	10.06991	−10.2473	1.56E-17	1.68E-15	29.37113	Down-Regulated
hsa-miR-324-3p	−1.36532	9.917577	−9.47768	8.45E-16	6.08E-14	25.42768	Down-Regulated
hsa-miR-720	−1.46921	12.4055	−8.86408	2.02E-14	1.02E-12	22.29872	Down-Regulated
hsa-miR-30d	−1.15751	13.04519	−8.42283	1.94E-13	9.15E-12	20.06631	Down-Regulated
hsa-miR-550	−1.67819	9.709234	−8.11338	9.40E-13	3.69E-11	18.51341	Down-Regulated
hsa-miR-132	−2.22146	4.350623	−7.72266	6.76E-12	2.16E-10	16.57212	Down-Regulated
hsa-miR-320b	−1.17778	11.65868	−7.62165	1.12E-11	3.46E-10	16.07431	Down-Regulated
hsa-miR-30c	−1.16476	11.15127	−6.68633	1.11E-09	2.51E-08	11.56764	Down-Regulated
hsa-miR-483-3p	−1.75494	5.540333	−6.5849	1.80E-09	3.78E-08	11.09214	Down-Regulated

### Functional Enrichment Analysis

**FIGURE 4:**
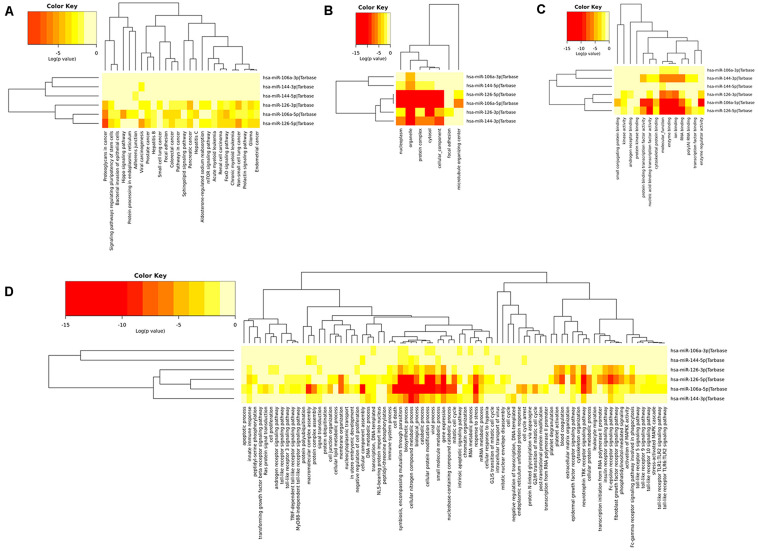
Functional enrichment of the target genes of the upmost 10 most up-regulated miRNAs. **(A)** enriched Kyoto encyclopedia of genes and genomes (KEGG) pathways of these target genes; **(B)** enriched cellular component (CC) of these target genes; **(C)** enriched molecular function (MF) of these target genes, **(D)** enriched biological process (BP) of these target genes.

**FIGURE 5:**
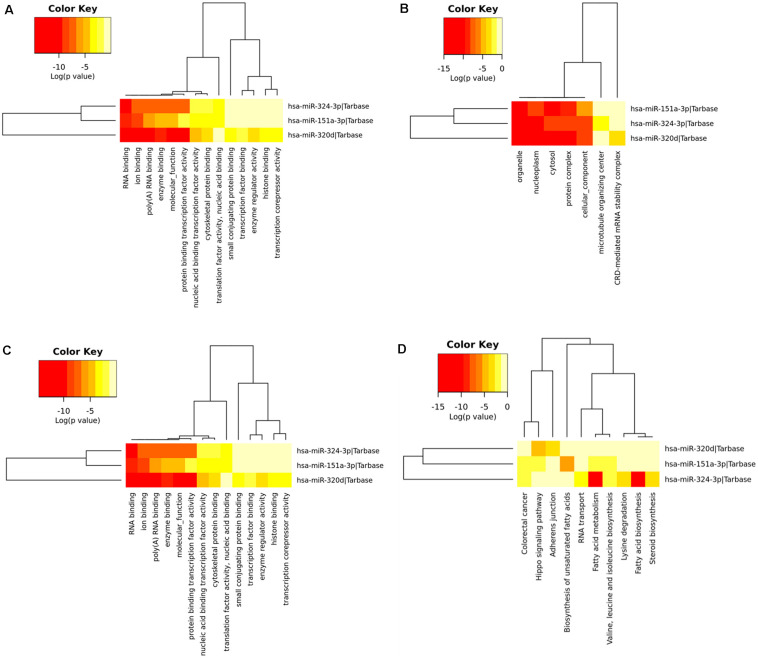
Functional enrichment of the target genes of the upmost ten most down-regulated miRNAs. **(A)** enriched Kyoto encyclopedia of genes and genomes (KEGG) pathways of these target genes; **(B)** enriched cellular component (CC) of these target genes; **(C)** enriched molecular function (MF) of these target genes; **(D)** enriched biological process (BP) of these target genes.

To determine the enriched pathways and process enrichment of these target genes, we afterward carried out a KEGG pathway enrichment and GO functional annotation. The KEGG pathway analysis included non-small cell lung cancer, proteoglycans in cancer, viral carcinogenesis, *etc.* (Figure 4A, Table 2). We selected 3 GO categories consisting of biological pathway (BP), cellular component (CC), along with molecular function (MF) for functional annotation. The top ten GO terms of the target genes of the 10 upmost up-regulated DE-miRNAs are indicated in Figures 4B–D and Table 3, including the responses to stress, catabolic process, gene expression, *etc.* in the BP category; cellular component, nucleoplasm, cytosol, *etc.* in the CC category; and enzyme binding, nucleic acid binding transcription factor activity, RNA binding, *etc.* in the MF category. Subsequently, the target genes of the ten upmost downregulated DE-miRNAs were analyzed in the same way, and the results are shown in Figures 5A–D and Tables 4, 5.

**TABLE 2:** KEGG pathway analysis of unregulated DE-miRNAs in sarcoidosis.

KEGG pathway	*p*-value	Genes	miRNAs
Proteoglycans in cancer (hsa05205)	7.77E-16	PIK3R2, PIK3CD, CTNNB1, AKT1, PIK3CA, SDC2, VEGFA, MAPK1, ITGB1, PTCH1, WNT5A, PIK3R2, FRS2, RDX, KRAS, FZD4, PIK3CD, CCND1, CTNNB1, AKT1, FLNB, GAB1, PIK3CA, SDC2, PDPK1, VEGFA, MAPK1, GRB2, MDM2, RPS6KB1, PDCD4, ITGB1, EZR, CBL, NRAS, CAV1, ROCK2, FRS2, IGF1R, RHOA, PPP1R12B, CAV2, FZD3, PPP1R12A, CCND1, CTNNB1, FLNB, TIMP3, PIK3R1, SOS1, DDX5, GAB1, AKT3, CDKN1A, VEGFA, MAPK1	hsa-miR-126-3p, hsa-miR-126-5p, hsa-miR-106a-5p
Viral carcinogenesis (hsa05203)	8.91E-08	CCND2, YWHAB, DDX3X, CASP3, JUN, EP300, USP7, HIST2H2BE, GTF2A1, PIK3R2, HIST1H2BD, PIK3CD, GTF2H3, CCNE1, PIK3CA, IL6ST, HDAC8, MAPK1, ATF2, RASA2, DLG1, PIK3R2, CDK1, KRAS, CDK6, PMAIP1, PIK3CD, CCND1, YWHAZ, HDAC2, RBPJ, ACTN4, CCNE1, PIK3CA, USP7, MAPK1, CREBBP, GRB2, JAK1, MDM2, CCND3	hsa-miR-144-3p, hsa-miR-126-3p, hsa-miR-126-5p
Non-small cell lung cancer (hsa05223)	4.87E-07	E2F1, PIK3R2, PIK3CD, E2F3, AKT1, PIK3CA, MAPK1, RXRA, PIK3R2, KRAS, CDK6, PIK3CD, CCND1, E2F3, AKT1, PIK3CA, PDPK1, MAPK1, GRB2, E2F1, NRAS, STK4, CCND1, PIK3R1, RB1, SOS1, AKT3, MAPK1, RASSF5	hsa-miR-126-3p, hsa-miR-126-5p, hsa-miR-106a-5p
Pancreatic cancer (hsa05212)	5.51E-06	E2F1, PIK3R2, PIK3CD, E2F3, AKT1, PIK3CA, VEGFA, MAPK1, PIK3R2, KRAS, CDK6, PIK3CD, CCND1, E2F3, AKT1, PIK3CA, VEGFA, MAPK1, TGFBR2, JAK1, STAT3, E2F1, SMAD3, CCND1, SMAD4, MAPK8, PIK3R1, RB1, AKT3, VEGFA, MAPK1, TGFBR2, JAK1	hsa-miR-126-3p, hsa-miR-126-5p, hsa-miR-106a-5p
Colorectal cancer (hsa05210)	9.75E-06	PIK3R2, PIK3CD, CTNNB1, AKT1, PIK3CA, MAPK1, GSK3B, PIK3R2, BCL2, KRAS, PIK3CD, CCND1, CTNNB1, AKT1, PIK3CA, MAPK1, TGFBR2, TCF7L1, SMAD3, RHOA, APPL1, CCND1, SMAD4, CTNNB1, MAPK8, PIK3R1, AKT3, LEF1, MAPK1, TGFBR2	hsa-miR-126-3p, hsa-miR-126-5p, hsa-miR-106a-5p
Glioma (hsa05214)	1.33E-05	PIK3R2, KRAS, CDK6, PIK3CD, CCND1, E2F3, AKT1, PIK3CA, MAPK1, GRB2, MDM2, PDGFRA, E2F1, NRAS, IGF1R, CCND1, PIK3R1, RB1, SOS1, AKT3, CDKN1A, MAPK1	hsa-miR-126-3p, hsa-miR-126-5p, hsa-miR-106a-5p
FoxO signaling pathway (hsa04068)	1.34E-05	IRS2, PIK3R2, STK11, PIK3CD, AKT1, PLK2, IRS1, PIK3CA, MAPK1, SGK1, IRS2, CCNB1, SIRT1, PIK3R2, KRAS, PIK3CD, CCND1, AKT1, IRS1, PRKAA1, BCL6, PRKAB2, SOD2, PIK3CA, USP7, PDPK1, MAPK1, CREBBP, GRB2, CCNG2, TGFBR2, MDM2, RBL2, STAT3, CCNB1, NRAS, STK4, SIRT1, SETD7, CCND2, STK11, SMAD3, IGF1R, NLK, CCND1, SMAD4, MAPK8, PLK2, PIK3R1, SOS1, IRS1, PRKAB2, CSNK1E, AKT3, SOD2, CDKN1A, MAPK1, CCNG2, TGFBR2	hsa-miR-126-3p, hsa-miR-126-5p,hsa-miR-106a-5p
Renal cell carcinoma (hsa05211)	1.64E-05	CRK, CUL2, PIK3R2, RAP1A, ETS1, KRAS, PIK3CD, AKT1, GAB1, PIK3CA, VEGFA, MAPK1, CREBBP, GRB2, NRAS, CRK, PAK2, ARNT, ETS1, EPAS1, EGLN3, PIK3R1, SOS1, GAB1, AKT3, VEGFA, MAPK1, EGLN1	hsa-miR-126-3p,hsa-miR-126-5p,hsa-miR-106a-5p
Chronic myeloid leukemia (hsa05220)	4.95E-05	E2F1, CRK, PIK3R2, CTBP2, PIK3CD, E2F3, AKT1, PIK3CA, MAPK1, KRAS, CDK6, CCND1, HDAC2, GRB2, TGFBR2, MDM2, CBL, NRAS, RUNX1, SMAD3, SMAD4, PIK3R1, RB1, SOS1, AKT3, CDKN1A	hsa-miR-126-3p, hsa-miR-126-5p, hsa-miR-106a-5p
Pathways in cancer (hsa05200)	5.12E-05	E2F1, CRK, PIK3R2, GNA13, ETS1, CTBP2, PIK3CD, CTNNB1, E2F3, AKT1, CCNE1, PIK3CA, ITGA6, VEGFA, MAPK1, ADCY9, RXRA, GSK3B, ITGB1, CRK, PTCH1, CUL2, WNT5A, PIK3R2, GNA13, ETS1, BCL2, KRAS, CDK6, FZD4, PIK3CD, CCND1, CTNNB1, E2F3, AKT1, HDAC2, CCNE1, PIK3CA, VEGFA, MAPK1, CREBBP, GRB2, TGFBR2, JAK1, MDM, STAT3, PDGFRA, E2F1, ITGB1, CXCL8, CBL, CXCR4, NRAS, STK4, CRK, RUNX1, ROCK2, TCF7L1, ARNT, ETS1, SMAD3, IGF1R, RHOA, APPL1, FZD3, TPM3, EPAS1, CCND1, SMAD4, CTNNB1, COL4A2, MAPK8, EGLN3, PIK3R1, RB1, SOS1, HSP90B1, AKT3, LEF1, CDKN1A, ITGA6, VEGFA, MAPK1, TGFBR2, JAK1, EGLN1, COL4A1, RASSF5	hsa-miR-126-3p, hsa-miR-126-5p, hsa-miR-106a-5p

**TABLE 3:** GO analysis of unregulated DE-miRNAs associated with sarcoidosis.

GO Category	Gene function (Term)	Gene count	miRNAs	*p*-value
BP	Response to stress (GO:0006950)	313	hsa-miR-144-3p, hsa-miR-126-3p, hsa-miR-126-5p, hsa-miR-106a-5p	<1e-325
BP	Catabolic process (GO:0009056)	249	hsa-miR-144-3p, hsa-miR-126-3p, hsa-miR-126-5p, hsa-miR-106a-5p	<1e-325
BP	Gene expression (GO:0010467)	119	hsa-miR-144-3p, hsa-miR-126-3p, hsa-miR-126-5p, hsa-miR-106a-5p	<1e-325
BP	Fc-epsilon receptor signaling pathway (GO:0038095)	38	hsa-miR-144-3p, hsa-miR-126-3p, hsa-miR-126-5p, hsa-miR-106a-5p	<1e-325
BP	Neurotrophin TRK receptor signaling pathway (GO:0048011)	58	hsa-miR-144-3p, hsa-miR-126-3p, hsa-miR-126-5p, hsa-miR-106a-5p	<1e-325
BP	Cellular protein modification process (GO:0006464)	376	hsa-miR-144-3p, hsa-miR-126-3p, hsa-miR-126-5p, hsa-miR-106a-3p, hsa-miR-106a-5p	<1e-325
BP	Biological_process (GO:0008150)	1711	hsa-miR-144-3p, hsa-miR-144-5p, hsa-miR-126-3p, hsa-miR-126-5p, hsa-miR-106a-5p	<1e-325
BP	Viral process (GO:0016032)	101	hsa-miR-144-3p, hsa-miR-126-3p, hsa-miR-126-5p, hsa-miR-106a-3p, hsa-miR-106a-5p	<1e-325
BP	Cellular nitrogen compound metabolic process (GO:0034641)	647	hsa-miR-144-3p, hsa-miR-144-5p, hsa-miR-126-3p, hsa-miR-126-5p, hsa-miR-106a-5p	<1e-325
BP	biosynthetic process (GO:0009058)	536	hsa-miR-144-3p,hsa-miR-144-5p, hsa-miR-126-3p, hsa-miR-126-5p, hsa-miR-106a-3p, hsa-miR-106a-5p	< 1e-325
CC	cellular component (GO:0005575)	1729	hsa-miR-144-3p, hsa-miR-126-3p, hsa-miR-126-5p, hsa-miR-106a-5p	< 1e-325
CC	nucleoplasm (GO:0005654)	196	hsa-miR-144-3p, hsa-miR-144-5p, hsa-miR-126-3p, hsa-miR-126-5p, hsa-miR-106a-5p	< 1e-325
CC	cytosol (GO:0005829)	419	hsa-miR-144-3p, hsa-miR-144-5p, hsa-miR-126-3p, hsa-miR-126-5p, hsa-miR-106a-5p	< 1e-325
CC	protein complex (GO:0043234)	483	hsa-miR-144-3p, hsa-miR-144-5p, hsa-miR-126-3p, hsa-miR-126-5p, hsa-miR-106a-5p	< 1e-325
CC	organelle (GO:0043226)	1324	hsa-miR-144-3p, hsa-miR-144-5p, hsa-miR-126-3p, hsa-miR-126-5p, hsa-miR-106a-3p, hsa-miR-106a-5p	< 1e-325
CC	microtubule organizing center (GO:0005815)	64	hsa-miR-126-5p, hsa-miR-106a-5p	3.37E-06
CC	focal adhesion (GO:0005925)	13	hsa-miR-126-3p	0.013148
MF	nucleic acid binding transcription factor activity (GO:0001071)	161	hsa-miR-144-3p, hsa-miR-126-3p, hsa-miR-126-5p, hsa-miR-106a-5p	< 1e-325
MF	RNA binding (GO:0003723)	266	hsa-miR-144-3p, hsa-miR-126-3p, hsa-miR-126-5p, hsa-miR-106a-5p	< 1e-325
MF	enzyme binding (GO:0019899)	233	hsa-miR-144-3p, hsa-miR-126-3p, hsa-miR-126-5p, hsa-miR-106a-3p, hsa-miR-106a-5p	< 1e-325
MF	ion binding (GO:0043167)	756	hsa-miR-144-3p, hsa-miR-126-3p, hsa-miR-126-5p, hsa-miR-106a-3p, hsa-miR-106a-5p	< 1e-325
MF	molecular_function (GO:0003674)	1767	hsa-miR-144-3p, hsa-miR-144-5p, hsa-miR-126-3p, hsa-miR-126-5p, hsa-miR-106a-3p, hsa-miR-106a-5p	< 1e-325
MF	protein binding transcription factor activity (GO:0000988)	90	hsa-miR-144-3p, hsa-miR-126-5p, hsa-miR-106a-5p	2.22E-16
MF	enzyme regulator activity (GO:0030234)	109	hsa-miR-126-3p, hsa-miR-126-5p, hsa-miR-106a-5p	3.56E-12
MF	poly(A) RNA binding (GO:0044822)	205	hsa-miR-144-3p, hsa-miR-126-5p, hsa-miR-106a-5p	6.95E-08
MF	cytoskeletal protein binding (GO:0008092)	102	hsa-miR-144-3p, hsa-miR-126-5p, hsa-miR-106a-5p	1.82E-07
MF	transcription factor binding (GO:0008134)	46	hsa-miR-144-3p, hsa-miR-126-5p	0.000592

**TABLE 4:** KEGG pathway analysis of downregulated DE-miRNAs in sarcoidosis.

KEGG pathway	*p*-value	Genes	miRNAs
Fatty acid biosynthesis (hsa00061)	<1e-325	FASN	hsa-miR-324-3p
Fatty acid metabolism (hsa01212)	1.12E-09	PTPLB, ELOVL5, SCD, ACACA, FASN, CPT2	hsa-miR-151a-3p, hsa-miR-324-3p
Hippo signaling pathway (hsa04390)	6.90E-05	APC, PPP2CA, PPP1CC, SNAI2, YWHAB, CCND1, SMAD4, PPP2CB, LATS1, PARD6B, PPP2CA, YWHAG, CCND2, GLI2, SMAD3, WWTR1, CDH1, CTNNB1, CSNK1E, WNT2, SMAD7, LATS1	hsa-miR-151a-3p, hsa-miR-320d
Biosynthesis of unsaturated fatty acids (hsa01040)	0.000417325	PTPLB, ELOVL5, SCD	hsa-miR-151a-3p
Colorectal cancer (hsa05210)	0.001949491	BRAF, APC, RHOA, PIK3R3, CCND1, SMAD4, MSH6, GSK3B, PIK3CB, BIRC5, CCND1, SMAD4, CTNNB1	hsa-miR-151a-3p, hsa-miR-324-3p
Adherens junction (hsa04520)	0.00509092	SMAD3, TJP1, CDH1, CTNNB1, RAC1, INSR, SSX2IP, MAPK1	hsa-miR-320d
RNA transport (hsa03013)	0.01706858	PAIP1, NUP160, EIF3B, NUP214, RNPS1, EIF2B3, EIF4B, EIF4G1, EEF1A1, NDC1, EIF4, BP2, NUP155, RAN, EIF4G2	hsa-miR-324-3p
Lysine degradation (hsa00310)	0.02839269	WHSC1L1, ALDH3A2, SETD2, ASH1L, KMT2C	hsa-miR-324-3p
Steroid biosynthesis (hsa00100)	0.02915267	DHCR24	hsa-miR-324-3p
Valine, leucine and isoleucine biosynthesis (hsa00290)	0.04059014	BCAT2	hsa-miR-151a-3p, hsa-miR-324-3p

**TABLE 5:** GO analysis of downregulated DE-miRNAs associated with sarcoidosis.

GO Category	Gene function (Term)	Gene count	miRNAs	*p*-value
BP	Cellular protein modification process (GO:0006464)	231	hsa-miR-151a-3p, hsa-miR-320d, hsa-miR-324-3p	<1e-325
BP	Biosynthetic process (GO:0009058)	363	hsa-miR-151a-3p, hsa-miR-320d, hsa-miR-324-3p	<1e-325
BP	Gene expression (GO:0010467)	110	hsa-miR-151a-3p, hsa-miR-320d, hsa-miR-324-3p	<1e-325
BP	Cellular nitrogen compound metabolic process (GO:0034641)	461	hsa-miR-151a-3p, hsa-miR-320d, hsa-miR-324-3p	<1e-325
BP	Symbiosis, encompassing mutualism through parasitism (GO:0044403)	72	hsa-miR-151a-3p, hsa-miR-320d, hsa-miR-324-3p	7.99E-14
BP	Viral process (GO:0016032)	65	hsa-miR-151a-3p, hsa-miR-320d, hsa-miR-324-3p	1.59E-13
BP	Mitotic cell cycle (GO:0000278)	57	hsa-miR-151a-3p, hsa-miR-320d, hsa-miR-324-3p	3.04E-12
BP	Cell death (GO:0008219)	104	hsa-miR-151a-3p, hsa-miR-320d, hsa-miR-324-3p	9.11E-12
BP	Neurotrophin TRK receptor signaling pathway (GO:0048011)	40	hsa-miR-151a-3p, hsa-miR-320d, hsa-miR-324-3p	4.37E-11
BP	Biological_process (GO:0008150)	1138	hsa-miR-151a-3p, hsa-miR-320d, hsa-miR-324-3p,	1.42E-10
CC	Cellular_component (GO:0005575)	1189	hsa-miR-151a-3p, hsa-miR-320d, hsa-miR-324-3p	<1e-325
CC	Nucleoplasm (GO:0005654)	157	hsa-miR-151a-3p, hsa-miR-320d, hsa-miR-324-3p	<1e-325
CC	Cytosol (GO:0005829)	279	hsa-miR-151a-3p, hsa-miR-320d, hsa-miR-324-3p	<1e-325
CC	Organelle (GO:0043226)	889	hsa-miR-151a-3p, hsa-miR-320d, hsa-miR-324-3p	<1e-325
CC	Protein complex (GO:0043234)	349	hsa-miR-151a-3p, hsa-miR-320d, hsa-miR-324-3p	<1e-325
CC	CRD-mediated mRNA stability complex (GO:0070937)	5	hsa-miR-320d	0.006458676
CC	Microtubule organizing center (GO:0005815)	20	hsa-miR-324-3p	0.00699504
MF	Protein binding transcription factor activity (GO:0000988)	73	hsa-miR-151a-3p, hsa-miR-320d, hsa-miR-324-3p	<1e-325
MF	Molecular_function (GO:0003674)	1174	hsa-miR-151a-3p, hsa-miR-320d, hsa-miR-324-3p	<1e-325
MF	RNA binding (GO:0003723)	223	hsa-miR-151a-3p, hsa-miR-320d, hsa-miR-324-3p	<1e-325
MF	Enzyme binding (GO:0019899)	144	hsa-miR-151a-3p, hsa-miR-320d, hsa-miR-324-3p	<1e-325
MF	Ion binding (GO:0043167)	478	hsa-miR-151a-3p, hsa-miR-320d, hsa-miR-324-3p	<1e-325
MF	Poly(A) RNA binding (GO:0044822)	195	hsa-miR-151a-3p, hsa-miR-320d, hsa-miR-324-3p	<1e-325
MF	Nucleic acid binding transcription factor activity (GO:0001071)	93	hsa-miR-151a-3p, hsa-miR-320d, hsa-miR-324-3p	7.76E-07
MF	Cytoskeletal protein binding (GO:0008092)	76	hsa-miR-151a-3p, hsa-miR-320d, hsa-miR-324-3p	1.46E-05
MF	Translation factor activity, nucleic acid binding (GO:0008135)	15	hsa-miR-151a-3p, hsa-miR-324-3p	0.000339
MF	Enzyme regulator activity (GO:0030234)	38	hsa-miR-320d	0.002755

### Development and Analysis of the PPI Network and miRNA-Target Network

**FIGURE 6:**
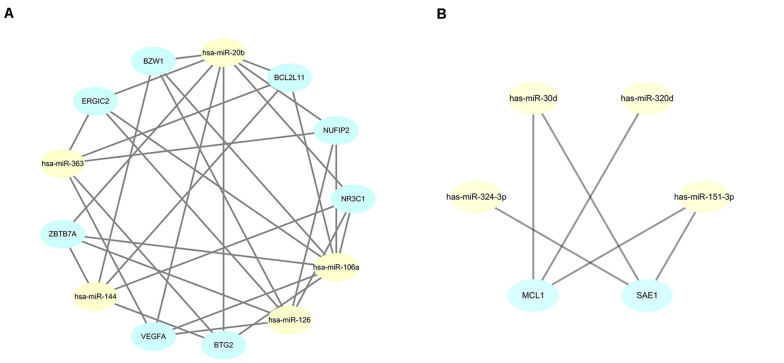
Simplified miRNA-gene network. **(A)** miRNA-gene network of upregulated miRNAs after simplified by interaction at degree ≥ 4; **(B)** miRNA-gene network of downregulated miRNAs after simplified by interaction at degree ≥ 3.

The STRING online data resource was used to develop the PPI network complex of the filtered target genes of the 5 upmost upregulated and downregulated miRNAs. By using Cystoscope software, the hub nodes were screened out and further developed the miRNA-hub gene network (Supplementary Figures 1, 2). After simplification, a concise relationship between miRNAs and hub genes was achieved. As shown in Figure 6A, *NR3C1*, *ZBTB7A*, *NUFIP2*, *BZW1, ERGIC2*, *VEGFA*, *BTG2*, and *BCL2L11* were the eight most targeted hub genes in the upregulation of miRNAs. Among these, four hub genes could potentially be modulated by the upregulation of hsa-miR-144. Six hub genes and eight hub genes could potentially be modulated by hsa-miR-126 and hsa-miR-106a, respectively. Eight and five hub genes could potentially be modulated by hsa-miR-20b and hsa-miR-363, respectively. Moreover, *MCL1* and *SAE1* were the two most targeted hub genes in the downregulation of miRNA. *MCL1* could potentially be targeted by hsa-miR-30d, hsa-miR-320d, and hsa-miR-151-3p; *SAE1* could potentially be regulated by hsa-miR-324-3p, hsa-miR-30d, as well as hsa-miR-151-3p (Figure 6B).

To further identify reliable hub genes, the targeted hub genes of miRNAs were screened in the gene nodes of the PPI network, and only two overlapping genes (*NR3C1*, *VEGFA*) were found with a relatively high degree (19 and 18, respectively) in the up and downregulated hub genes. After combining the above results, *NR3C1* and *VEGFA* were selected and potentially modulated by hsa-miR-126, hsa-miR-20b, and hsa-miR-106a. Of note, these three miRNAs might be potential regulators in manipulating the pathogenesis and development of sarcoidosis.

### Verification of the Expression of Hub Genes and miRNAs in Clinical Specimens

**FIGURE 7:**
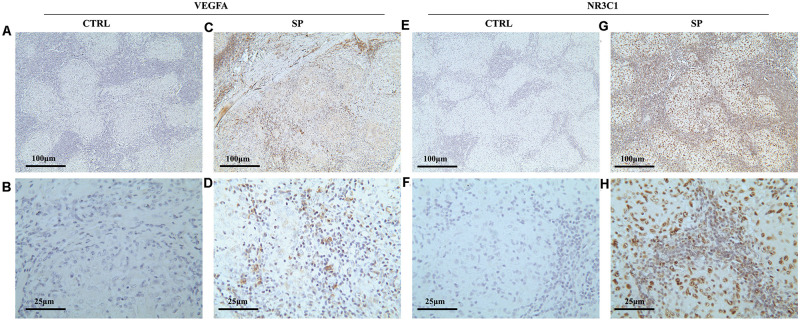
Immunohistochemical methods were employed to assess the cellular localization and expression levels of NR3C1 and VEGFA in mediastinal lymph node tissues of pulmonary sarcoidosis (SP group, *n* = 12) and tuberculosis (CRTL group, *n* = 13). **(A–D)** VEGFA (+); **(E–H)** NR3C1 (+). (10×: ACEG; 40×: BDFH).

To validate the expression of *VEGFA* and *NR3C1*, we performed IHC to examine 12 mediastinal lymph node tissues from pulmonary sarcoidosis patients and 13 mediastinal lymph node tissues from tuberculosis patients. Ten of the twelve sections showed moderate to strong dark brown staining of VEGFA in both the epithelioid cells and lymphocytes (Figures 7C,D), and negative to very weak staining for control group (Figures 7A,B). Nine of the twelve sections presented weak to moderate staining expression of NR3C1 (Figures 7G,H) comparing the control sections which present negative to very weak staining (Figures 7E,F).

**FIGURE 8:**
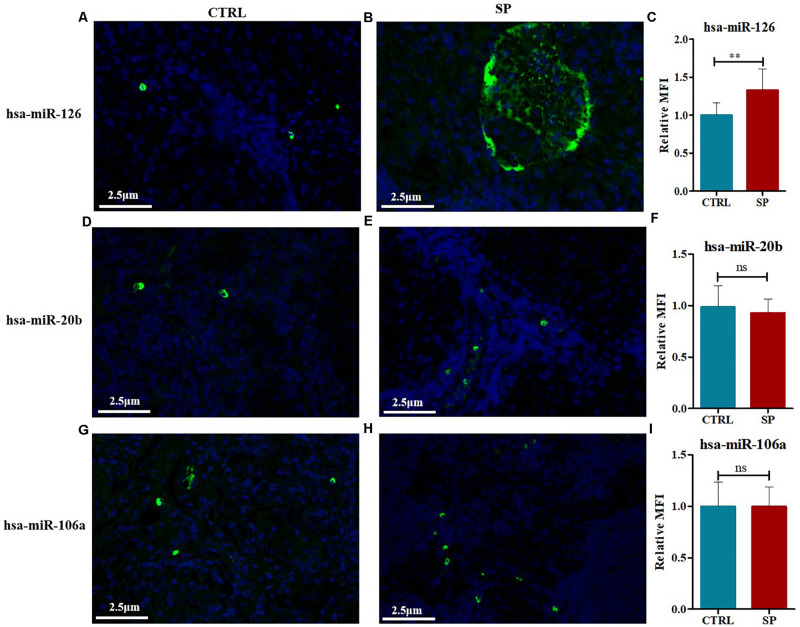
FISH methods were conducted to detect the expression levels of hsa-miR-126, hsa-miR-20b, as well as hsa-miR-106a in mediastinal lymph node tissues of pulmonary sarcoidosis (SP group, *n* = 12, 400×) and tuberculosis (CRTL group, *n* = 13, 400×). **(A–C)** hsa-miR-126 (+); **(D–F)** hsa-miR-20b (+); **(G–I)**: hsa-miR-106a (+). Data have been represented as mean ± SD, **p < 0.01. MFI: mean fluorescence; ns: no significant.

The expression contents of hsa-miR-126, hsa-miR-20b, and hsa-miR-106a were further analyzed via FISH on the 12 mediastinal lymph node tissues of pulmonary sarcoidosis patients versus the 13 control tissues. Typical results were shown in Figure 8. The staining revealed strong enrichment of hsa-miR-126 along the edge of the non-caseating granulomas (Figure 8B), presenting higher expression than the controls (Figure 8A), while hsa-miR-20b (Figures 8D,E) and hsa-miR-106a had low expression (Figures 8G,H) in both groups. Quantification of fluorescence for hsa-miR-126, hsa-miR-20b, and hsa-miR-106a were measured, and it demonstrated that hsa-miR-126 significantly increased in mediastinal lymph node tissues of pulmonary sarcoidosis patients (Figures 8C,F,I).

## Discussion

Sarcoidosis, a multisystem disease, is typified by non-caseating granulomas. Up to 50% of sarcoidosis cases are diagnosed by chest radiographs during a routine examination. Clinically, approximately 1/4 of pulmonary sarcoidosis cases are atypical with non-specific clinical symptoms, atypical radiological manifestations, and non-unique pathology, which easily lead to misdiagnosis (Aryal and Nathan, 2019). The etiology and pathogenesis are still uncertain. Although intensive studies have revealed genetic factors, inflammation, and immune responses in the risk and clinical development of sarcoidosis, the regulation of miRNAs is rarely involved. Our study indicated that multiple complex signal transduction pathways and factors participate in the pathogenic mechanisms of sarcoidosis. The microarray and bioinformatics analysis provides us with a comprehensive map of the biological functions and interactions of sarcoidosis-related genes, this will not only provide us with pivotal information to reveal the pathogenesis of the disease, but also help us to find novel preventive and therapeutic interventions.

Herein, we explored the gene expression profile of GSE26409 consisting of 45 sarcoidosis blood samples and 55 normal blood samples, to identify the molecular mechanism of sarcoidosis. Totally,266 DE-miRNAs were filtered, consisting of 132 upregulated and 134 downregulated miRNAs. Among these, hsa-miR-144, hsa-miR-126 along with hsa-miR-106a were the most upregulated miRNAs; hsa-miR-151-3p, hsa-miR-320d, and hsa-miR-324-3p were the most downregulated miRNAs. At the molecular level, prediction of the miRNA target genes was performed, then GO annotation and associated cascades were analyzed. Furthermore, by constructing the protein–protein interaction (PPI) networks, *NR3C1*, *ZBTB7A*, *NUFIP2*, *BZW1, ERGIC2*, *VEGFA*, *BTG2*, and *BCL2L11* were mapped as the most targeted hub genes in the upregulation of miRNAs, and *MCL1* and *SAE1* were the most targeted hub genes in the downregulation of miRNA. *NR3C1* and *VEGFA* were selected and are potentially modulated by hsa-miR-126, hsa-miR-20b, and hsa-miR-106a. To validate the bioinformatics analysis data, immunohistochemistry along with fluorescence *in situ* hybridization were employed to assess gene expression in clinical specimens. Among the identified miRNAs involved in sarcoidosis, hsa-miR-126 was highly expressed, and the two target genes VEGFA and NR3C1 were also highly expressed in lymph node samples of sarcoidosis.

The GO annotation and KEGG pathway analysis have identified more cancer related pathways that were closely related to upregulated DE-miRNAs in sarcoidosis, which including proteoglycans in cancer, viral carcinogenesis, non-small cell lung cancer, pancreatic cancer, colorectal cancer, glioma, etc. The relationship between sarcoidosis and cancer is complex, and meta-analyses have concluded that an increased risk of neoplasia in sarcoidosis (El Jammal et al., 2020). Fatty acid biosynthesis and metabolism also participated in the biological functions of miRNA in sarcoidosis. The mismatch of myocardial fatty acid metabolism and myocardial perfusion under positron emission tomography computed tomography (PET-CT) could diagnosis and detection of myocardial injury in cardiac sarcoidosis (Momose et al., 2015).

VEGFA (Vascular endothelial growth factor-A) is a PDGF/VEGF growth factor family member, and by triggering the proliferation, as well as the migration of vascular endothelial cells, it is vital for physiological and pathological of angiogenesis (Tamura et al., 2019). A previous clinical study confirmed that the serum levels of VEGF were higher in sarcoidosis subjects than in normal controls (Mirsaeidi et al., 2018). Further research evaluated the VEGFA level in serum, as well as bronchoalveolar lavage (BAL) and found similar results: VEGFA was remarkably higher in individuals with sarcoidosis relative to the healthy controls (*p* = 0.0002). The results also showed that the immunoexpression of VEGFA was higher in serum in contrast to the BAL fluid. However, the levels of VEGFA had no correlation with the clinical phenotype (acute onset or insidious onset), stage, or lung function (Piotrowski et al., 2015). Immunohistochemistry analyses on VATS (video-assisted thoracoscopic surgery) lung biopsy samples of sarcoidosis lesions were very consistent with our findings that VEGF was upregulated compared to the control group (Tzouvelekis et al., 2012).

NR3C1 (Nuclear receptor subfamily 3 group C member-1) codes for the glucocorticoid receptor and extensively participates in cellular proliferation, differentiation of target tissues, as well as inflammatory responses. Mutations that occurred in NR3C1 are related to generalized glucocorticoid resistance. Glucocorticoids (GCs) are the most common therapeutic agents in clinical use, and due to the fundamental regulatory roles in suppression of inflammation, GCs were widely used for autoimmune and inflammatory diseases (Gharesouran et al., 2018). GWAS (Genome-wide association studies) have found that SNPs (single nucleotide polymorphisms) located in *NR3C1* were positively replicated in the corticosteroid drug pathway of asthma (Keskin et al., 2019). After evaluating the SNPs of *NR3C1* in *in vivo* and *in vitro* models, the results showed that it was closely associated with hypersensitivity to glucocorticoids in asthma (Cuzzoni et al., 2012). In this study, *NR3C1* was upregulated in sarcoidosis and expressed in pathological specimens, which may be related to the sensitivity of sarcoidosis to glucocorticoid therapy. Simultaneously, we found that NR3C1 is not always highly expressed, which may be related to the insensitivity of some sarcoidosis patients to glucocorticoid treatment in clinical practice. It can finally be seen that the DNA polymorphism reflects the heterogeneity of the disease.

At present, the role of miRNA in sarcoidosis remains unclear. Kiszałkiewicz et al. investigated the expression of ten screened miRNAs in 94 pulmonary sarcoidosis patients and 50 controls, and remarkable differences were discovered between the sarcoidosis patients and healthy controls in the expression of diverse detected miRNAs in peripheral blood lymphocytes (miR-27b, miR-let-7f, miR-15b, miR-192, miR-130a miR-221, miR-222). Three miRNAs were merely reported to differ in BALF cells (miR-15b, miR-192, as well as miR-221) (Kiszałkiewicz et al., 2016). A subgroup analysis further observed several correlations among the levels of expression of miRNA, lung function parameters (FVC, FEV_1_/FVC), and selected laboratory molecular markers (CD4+/CD8+, serum Ca^2+^ concentration). The results showed only gender was not related to the expression of miRNA in BALF (Kiszałkiewicz et al., 2016). Nevertheless, in our study, we did not evaluate the relationship of some clinical indicators to miRNAs expression. We summarized the following reasons. Firstly, the expression of miRNA in peripheral blood is affected by various factors throughout the body, so is the alveolar lavage fluid. For patients with lung infection, COPD, asthma, and tumors, the detected miRNA itself is not accurate enough, subsequently, error in correlation analysis is even greater. Secondly, for early stage sarcoidosis, the lung function is hardly affected, especially FVC, FEV1/FVC, and diffusion function parameters. And, changes in lung function cannot reflect the true situation of patients with sarcoidosis. Thirdly, our study is a retrospective study, not all included patients had lung function tests and samples of alveolar lavage fluid, thus, some of these parameters could not be tested.

To elucidate the function of miRNAs in sarcoidosis, Jazwa et al. (2015) compared the expression levels of selected inflammatory miRNAs in patients with sarcoidosis and healthy individuals. After screening the miRNA transcriptome, miR-34a was isolated from sarcoidosis patients in peripheral blood mononuclear cells, which suggested that it may be involved in the pathology of sarcoidosis. Additionally, miR-155 was shown to be one of the miRNAs that are differentially expressed in macrophages, which are considered to have a role in formation of granuloma in sarcoidosis (Zhang et al., 2013). A study on the mechanism of miRNA in pulmonary sarcoidosis proved that miR-34a along with miR-155 enhance the secretion of interferon-gamma and negatively modulate SIRT-1, therefore inhibiting NF-κB transcription, as well as deacetylate the p53 protein, ultimately inactivating p53-regulated transcription and apoptosis (Kachamakova-Trojanowska et al., 2018). On the other hand, by establishing a murine model, Barna et al. (2016) found that elevated miRNA-33 promoted the formation of granuloma through the inhibition of the alveolar macrophage lipid transporters ABCA1 and ABCG1.

In our study, the lymphatic tissue of tuberculosis patients was used as a control group. This is why we raise the question from clinic and return to clinic through this research. Pulmonary sarcoidosis and tuberculosis are the most difficult to distinguish in clinical practice. Even if they have the gold standard of pathological diagnosis, it still needs experienced pathologists to give a definite diagnosis. Sometimes, for the small tissue, it can interfere with the definitive diagnosis of sarcoidosis. At the same time, the treatments for these two diseases are completely opposite. For treatment of sarcoidosis requires glucocorticoids, but the use of glucocorticoids for tuberculosis often causes the spread of disease. More significantly, the mediastinal lymph node of healthy people is not enlarged, and it is not available or difficult to obtain, which also violates ethical principles. Therefore, we used lymphatic tissue of tuberculosis as control group in this study.

There are still several limitations in this study. First of all, in the section of clinical specimen verification, the sample size we included is still small. Although immunohistochemistry and FISH have obtained positive results, a large sample study is still needed to further corroborate our results. Secondly, the specific regulatory mechanism of miR-126 in the pathogenesis and development of sarcoidosis still needs to be explored *in vitro* and *in vivo*. Finally, the incidence of sarcoidosis in different races is slightly different. In this study, we only included Asian individuals. This may bring certain limitations to our results.

From our results, we found that VEGFA and NR3C1 were overexpressed in the pathological tissue of sarcoidosis, which was mainly regulated by hsa-miR-126. To our knowledge, miRNAs are small non-coding RNAs that function as negative gene expression regulators. However, emerging evidences have shown that its function was switching from repression to activation, and its gene-activation function can up-regulate translation in nuclear (Vasudevan et al., 2007; Xiao et al., 2017). The GO annotation of our study comprised the molecular function on current biological knowledge of miRNA in sarcoidosis, which showed nucleic acid binding transcription factor activity has a pronounced gene count. This novel finding was also worth exploring for the study of the role of miRNAs in the regulation of sarcoidosis.

In summary, this work sheds further light on understanding the pathogenesis of sarcoidosis and might indicate novel diagnostic markers and treatment targets in the clinical practice of sarcoidosis. Nevertheless, further studies should be conducted to clarify the role of miRNAs and their related pathways in the pathogenesis of sarcoidosis.

## Data Availability Statement

The original contributions presented in the study are included in the article/Supplementary Material, further inquiries can be directed to the corresponding author/s.

## Ethics Statement

The studies involving human participants were reviewed and approved by Biomedical Ethics Committee of Second Affiliated Hospital of Xi’an Jiaotong University. The patients/participants provided their written informed consent to participate in this study.

## Author Contributions

YC, HZ, and QL performed the experiments, contributed to data analyses, as well as wrote the manuscript. YC, HZ, and LZ conceptualized the study design, contributed equally to data analyses and experimental materials. All authors contributed to the article and approved the submitted version.

## Conflict of Interest

The authors declare that the research was conducted in the absence of any commercial or financial relationships that could be construed as a potential conflict of interest.

## Footnotes

1 http://www.ncbi.nlm.nih.gov/geo

2 http://string-db.org/

## Supplementary Material

Supplementary Figure 1 miRNA-gene network of upregulated miRNAs (before simplified).

Supplementary Figure 2 miRNA-gene network of downregulated miRNAs (before simplified).
