# Supplementary figures and images for: Identification of the Core MicroRNAs and Potential Molecular Mechanismsin Sarcoidosis Using Bioinformatics Analysis
Source: Front Mol Biosci. 2021 May 13;8:644232. doi: 10.3389/fmolb.2021.644232 (PMC8155597; doi:10.3389/fmolb.2021.644232)

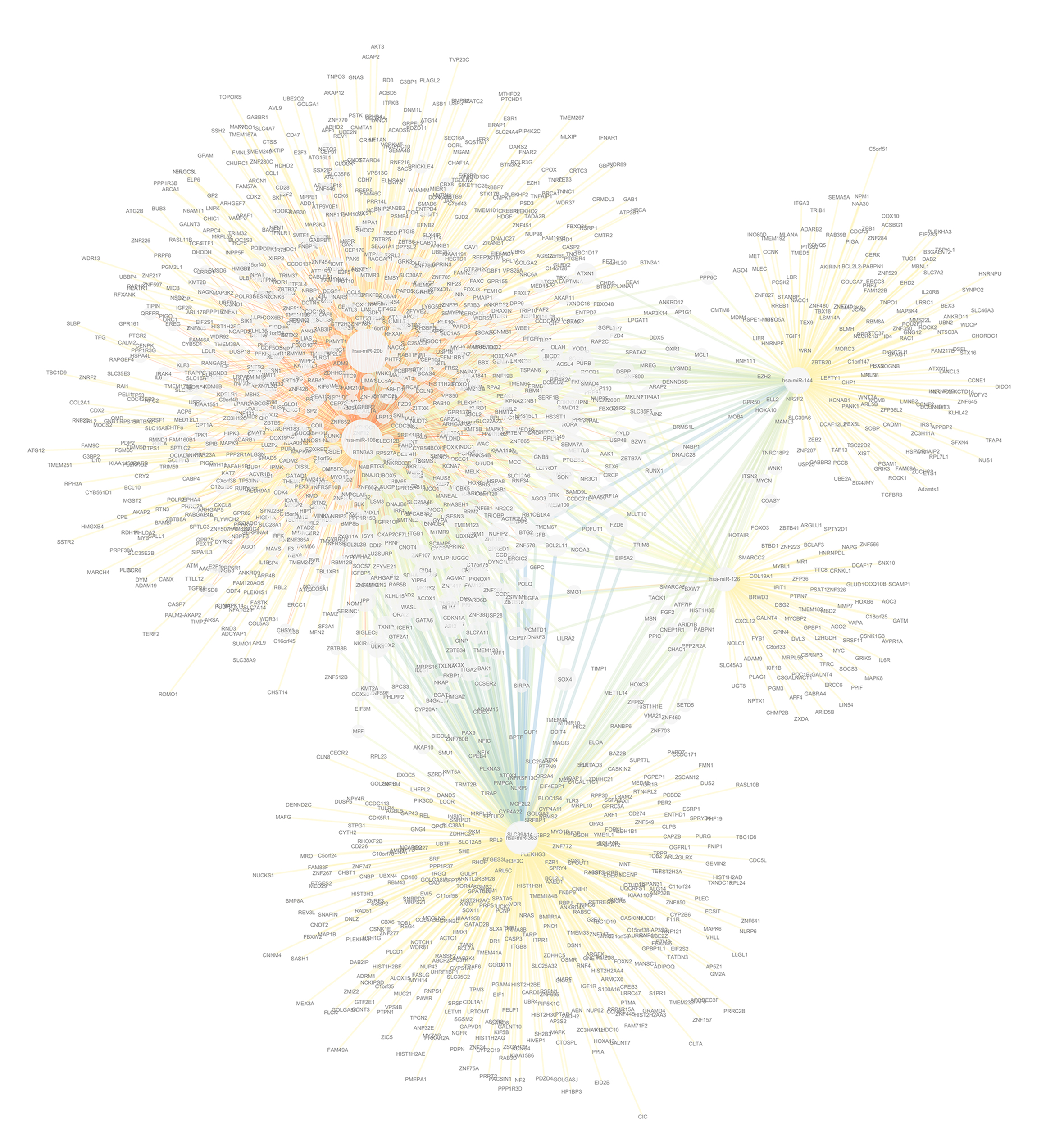

Supplement: Supplementary Figure 1 — miRNA-gene network of upregulated miRNAs (before simplified). [file Image_1.JPEG]

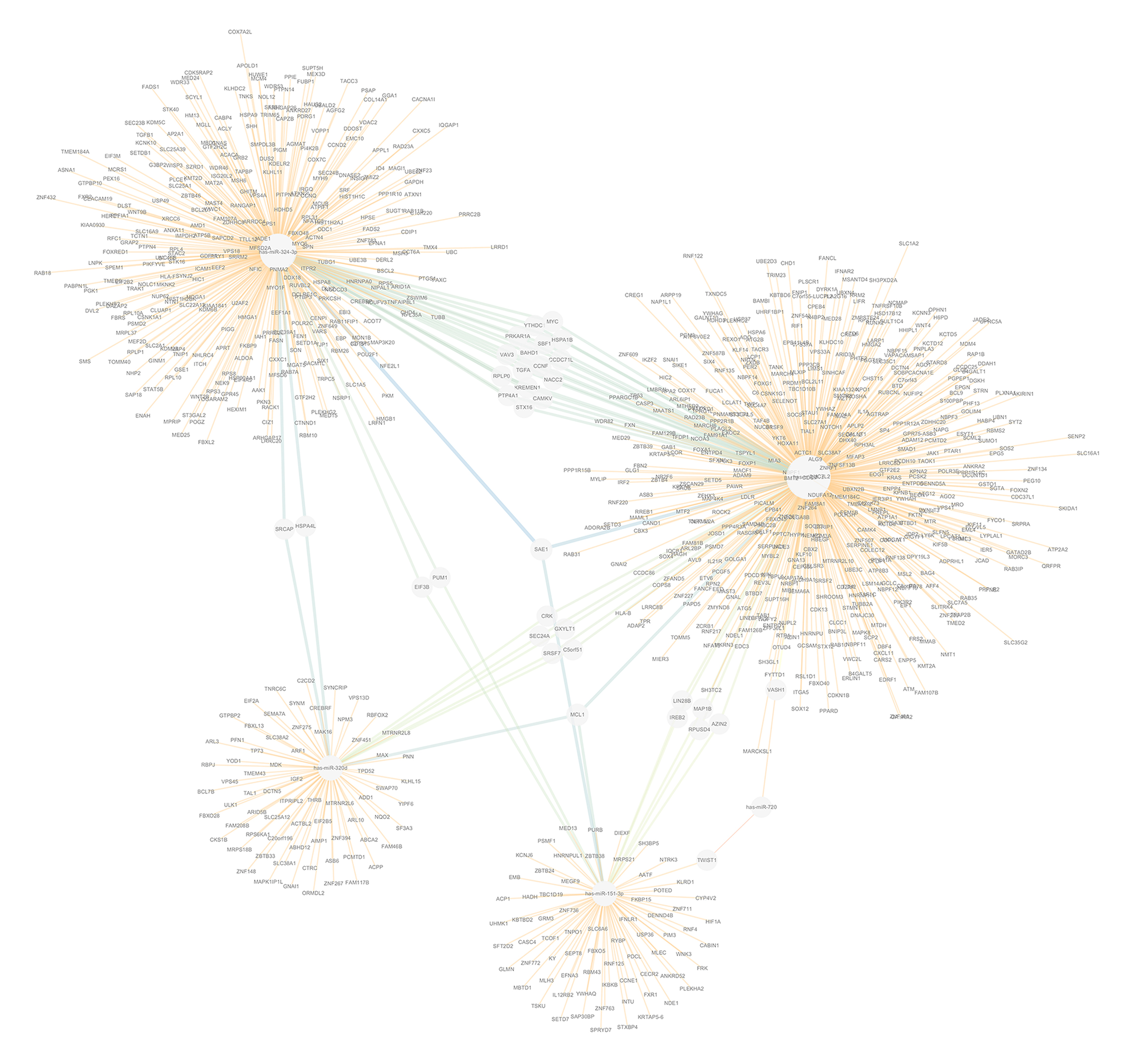

Supplement: Supplementary Figure 2 — miRNA-gene network of downregulated miRNAs (before simplified). [file Image_2.JPEG]
